# Comparison of Examination-Based and Self-Reported Risk Factors for Cardiovascular Disease, Washington State, 2006–2007

**DOI:** 10.5888/pcd9.110321

**Published:** 2012-06-21

**Authors:** Juliet Van Eenwyk, Lillian Bensley, Eric M. Ossiander, Karen Krueger

**Affiliations:** Author Affiliations: Lillian Bensley, Eric M. Ossiander, Karen Krueger, Washington State Department of Health, Olympia, Washington.

## Abstract

**Introduction:**

Obesity, hypertension, and high cholesterol are risk factors for cardiovascular disease, which accounts for approximately 20% of deaths in Washington State. For most states, self-reports from the Behavioral Risk Factor Surveillance System (BRFSS) provide the primary source of information on these risk factors. The objective of this study was to compare prevalence estimates of self-reported obesity, hypertension, and high cholesterol with examination-based measures of obesity, hypertension, and high-risk lipid profiles.

**Methods:**

During 2006–2007, the Washington Adult Health Survey (WAHS) included self-reported and examination-based measures of a random sample of 672 Washington State residents aged 25 years or older. We compared WAHS examination-based measures with self-reported measures from WAHS and the 2007 Washington BRFSS (WA-BRFSS).

**Results:**

The estimated prevalence of obesity from WA-BRFSS (27.1%; 95% confidence interval [CI], 26.3%–27.8%) was lower than estimates derived from WAHS physical measurements (39.2%; 95% CI, 33.6%–45.1%) (*P* < .001). Prevalence estimates of hypertension based on self-reports from WA-BRFSS (28.1%; 95% CI, 27.4%–28.8%) and WAHS (33.4%; 95% CI, 29.4%–37.7%) were similar to the examination-based estimate (29.4%; 95% CI, 25.8%–33.4%). Prevalence estimates of high cholesterol based on self-reports from WA-BRFSS (38.3%; 95% CI, 37.5%–39.2%) and WAHS (41.8%; 95% CI, 35.8%–48.1%) were similar; both were lower than the examination-based WAHS estimate of high-risk lipid profiles (59.2%; 95% CI, 54.2%–64.2%) (*P* < .001).

**Conclusion:**

Self-reported heights and weights underestimate the prevalence of obesity. The prevalence of self-reported high cholesterol is significantly lower than the prevalence of high-risk lipid profiles. Periodic examination-based measurement provides perspective on routinely collected self-reports.

## MEDSCAPE CME

Medscape, LLC is pleased to provide online continuing medical education (CME) for this journal article, allowing clinicians the opportunity to earn CME credit.

This activity has been planned and implemented in accordance with the Essential Areas and policies of the Accreditation Council for Continuing Medical Education through the joint sponsorship of Medscape, LLC and Preventing Chronic Disease. Medscape, LLC is accredited by the ACCME to provide continuing medical education for physicians. 

Medscape, LLC designates this Journal-based CME activity for a maximum of 1 **AMA PRA Category 1 Credit(s)™**. Physicians should claim only the credit commensurate with the extent of their participation in the activity.

All other clinicians completing this activity will be issued a certificate of participation. To participate in this journal CME activity: (1) review the learning objectives and author disclosures; (2) study the education content; (3) take the post-test with a 70% minimum passing score and complete the evaluation at www.medscape.org/journal/pcd (4) view/print certificate.

**Release date: June 20, 2012; Expiration date: June 20, 2013**

### Learning Objectives

Upon completion of this activity, participants will be able to:

- Compare self-report and examination-based data regarding obesity
- Compare self-report and examination-based data regarding hypertension
- Compare self-report and examination-based data regarding hyperlipidemia
- Distinguish factors associated with undiagnosed hyperlipidemia

**CME EDITOR**

Ellen Taratus, Editor, *Preventing Chronic Disease*. Disclosure: Ellen Taratus has disclosed no relevant financial relationships.

**CME AUTHOR**

Charles P. Vega, MD, Health Sciences Clinical Professor; Residency Director, Department of Family Medicine, University of California, Irvine. Disclosure: Charles P. Vega, MD has disclosed no relevant financial relationships.

**AUTHORS AND CREDENTIALS**

Disclosures: Juliet Van Eenwyk, PhD; Lillian Bensley, PhD; Eric M. Ossiander, PhD; Karen Krueger, MBA, MN have disclosed no relevant financial relationships.

Affiliations: Juliet Van Eenwyk, PhD; Lillian Bensley, PhD; Eric M. Ossiander, PhD; Karen Krueger, MBA, MN, Washington State Department of Health, Olympia, Washington.

## Introduction

Obesity, hypertension, and high cholesterol are well-established as risk factors for cardiovascular disease. They are targets for public health efforts to reduce illness and death from ischemic heart disease and stroke, which account for about one-fifth of deaths in Washington State and in the United States (1). Although the National Health and Nutrition Examination Survey (NHANES) (2) provides national examination-based measures of these risk factors, knowledge about their prevalence and distribution at the state level is limited and based primarily on self-reports from the Behavioral Risk Factor Surveillance System (BRFSS)(3).

A few studies have compared self-reports and examination-based measures of risk factors for cardiovascular disease. These studies generally show that self-reports underestimate obesity prevalence (4-7). Studies have also found underestimation of the prevalence of high cholesterol (5,8,9) and hypertension (4,5,9) based on self-reports, but a recent study in New York City had mixed results (6).

We were unable to identify studies that compare self-reported high cholesterol with measures of high-risk lipid profiles; high-risk profiles are those that identify people who take medication to regulate blood cholesterol or have abnormal values for any cholesterol component or for triglycerides. Although medical science has advanced in understanding the roles of cholesterol components and triglycerides in the development of cardiovascular disease, researchers have not assessed how high-risk lipid profiles compare with self-reported high cholesterol.

The objective of this study was to compare prevalence estimates of self-reported obesity, hypertension, and high cholesterol with examination-based measures of obesity, hypertension, and high-risk lipid profiles.

## Methods

### Data sources

The Washington State Department of Health designed the Washington Adult Health Survey (WAHS) primarily to estimate the statewide prevalence of hypertension and high-risk lipid profiles and to determine whether these differed for people living in households that have an annual income of less than $35,000 compared with households that have higher incomes. WAHS used a 3-stage stratified cluster design, randomly selecting block groups stratified by median household income (<$25,000, $25,000–$34,999, and ≥$35,000), housing units in block groups, and 1 adult aged 25 or older in each housing unit. WAHS included adults who spoke English or Spanish, lived in the sampled residence at least half the year, and were their own legal guardians. WAHS excluded pregnant women and people who had hemophilia or were being treated for cancer.

Field personnel included nurses and interviewers. Study procedures took place in participants’ homes. The first home visit included recruitment; informed consent; and directions for fasting, completing self-administered questionnaires, and having containers of prescription medications available at the next visit. The second visit included interviews on medical conditions, collection of fasting blood samples, physical measurements, and review of prescription medication containers. The interviews included asking participants, “Have you taken any prescription medicine in the past 30 days?” For those answering yes, the nurse said, “I would like to look at the medicine containers or packages to record what they are. Do you have your medicines available?” Participants received a $45 Visa debit card and information about their blood pressure, blood glucose and lipids, and body mass index (BMI). Data were collected from August 2006 to November 2007. 

Of 1,534 people determined to be eligible, 672 participated in WAHS, a participation rate of 44%. The Council of American Survey Research Organizations (CASRO) response rate (10), which also accounts for people not reached or for whom eligibility is not determined, was 38% (11). 

We also used self-reported data from the 2007 Washington State (WA) BRFSS (12), excluding participants younger than 25 years and pregnant women to maximize comparability with WAHS. The 2007 WA-BRFSS was a random-digit–dialed telephone survey of English and Spanish-speaking noninstitutionalized Washington state residents who lived in households with landline telephones. The CASRO response rate for the 2007 WA-BRFSS was 45% (13). The Washington State Institutional Review Board approved all WAHS and WA-BRFSS procedures. 

### Measures

#### Obesity

This study defined obesity as BMI of 30 kg/m^2^ or more, overweight as BMI of 25 kg/m^2^ to less than 30 kg/m^2^, and neither overweight nor obese as BMI of less than 25 kg/m^2^. The WAHS examination-based measure of obesity was computed from measured heights and weights following protocols adapted from NHANES (14). The WA-BRFSS self-reported measure of obesity was computed from responses to questions asking, “About how tall are you without shoes?” and “About how much do you weigh without shoes?”

#### Hypertension

A WAHS nurse measured blood pressure using NHANES protocols (15). Because blood pressure measured on 1 occasion is not sufficient for clinical diagnosis of hypertension, this study used a range of definitions reflecting levels of diagnostic certainty to estimate the prevalence of hypertension among WAHS participants. Categories are mutually exclusive so that participants classified at higher levels of certainty are not considered for lower levels. Participants were classified as having definite hypertension if the nurse noted containers for medications used only to control hypertension. Participants were classified as having probable hypertension if they had measured systolic blood pressure of 140 mm Hg or more or diastolic blood pressure of 90 mm Hg or more, or they reported that a health care provider had said they had high blood pressure on more than 1 occasion and they also had containers for medications often used to control blood pressure but could be used for other conditions. Participants were classified as having possible hypertension if they reported that a health care provider said they had high blood pressure on at least 1 occasion and they reported using medication for high blood pressure. Participants who reported that a health care provider said they had high blood pressure on at least 1 occasion were classified as probable no. Participants who reported none of the above were classified as definite no.

The primary examination-based hypertension measure included participants who had definite or probable hypertension. This study also included a secondary examination-based measure of hypertension similar to that used by NHANES (16): measured systolic blood pressure of 140 mm Hg or more, measured diastolic blood pressure of 90 mm Hg or more, or self-reported use of medication to control blood pressure based on answering yes to questions asking whether they had been told to take prescribed medicine and whether they were following this advice.

This study coded WAHS and WA-BRFSS participants as self-reporting hypertension if they answered yes to a question asking whether a health care provider had ever told them that they had high blood pressure.

#### High cholesterol and high-risk lipid profiles

WAHS nurses collected fasting blood samples and then processed and shipped the samples according to protocols of the Northwest Lipid Metabolism and Diabetes Research Center. The center’s laboratory (a participant in the Centers for Disease Control and Prevention lipid standardization program) determined levels of total cholesterol; high-density lipoprotein (HDL) and low-density lipoprotein (LDL) cholesterol; and triglycerides. Very low-density lipoprotein (VLDL) cholesterol was calculated for participants with triglycerides of 400 mg/dL or less by subtracting HDL and LDL values from total cholesterol. Abnormal lipid values were defined as total cholesterol of 240 mg/dL or more, LDL cholesterol of 160 mg/dL or more, VLDL cholesterol of 40 mg/dL or more, HDL cholesterol of less than 40 mg/dL for men and less than 50 mg/dL for women, or triglycerides of 200 mg/dL or more.

Because lipid values measured on 1 occasion are not sufficient for clinical diagnosis, this study used a range of definitions reflecting levels of diagnostic certainty to estimate the prevalence of high-risk lipid profiles among WAHS participants. Categories are mutually exclusive so that participants classified at higher levels of certainty are not considered for lower levels. Participants were classified as having definite high-risk lipid profiles if the nurse recorded that they were taking medications to control blood cholesterol. They were classified as having probable high-risk lipid profiles if they had at least 1 abnormal lipid value. Participants who had at least 1 borderline lipid value (total cholesterol of 200–239 mg/dL; LDL cholesterol of 100–159 mg/dL; VLDL cholesterol of 30–39 mg/dL; HDL cholesterol of 40–44 mg/dL for men or 50–54 mg/dL for women; or triglycerides of 150–199 mg/dL) or reported using medication or diet change to lower cholesterol were classified as possible high-risk lipid profile. Participants who reported that a health care provider said they had high blood cholesterol on at least 1 occasion were classified as probable no. Participants who reported none of the above were classified as definite no. Participants classified with definite or probable high-risk lipid profiles were coded as having high-risk lipid profiles.

This study coded WAHS and WA-BRFSS participants as self-reporting high cholesterol if they answered yes to “Have you ever had your blood cholesterol checked?” and “Have you ever been told by a doctor or other health professional that your blood cholesterol level was high?”; participants were coded as not self-reporting high cholesterol if they answered yes to the first and no to the second question. We excluded from this comparison participants who said they had not had their cholesterol checked.

#### Possible undiagnosed hypertension and high-risk lipid profiles

This study coded WAHS participants as having possible undiagnosed hypertension if they did not report ever being told by a health care provider that they had high blood pressure, were not on medications used only to control blood pressure, and had measured systolic blood pressure of 140 mm Hg or more or diastolic blood pressure of 90 mm Hg or more. They were coded as having possible undiagnosed high-risk lipid profiles if they did not report ever being told that they had high cholesterol (including those never tested), were not on medications used to control cholesterol, and had at least 1 abnormal lipid value.

### Data analysis

This study provides prevalence estimates and 95% confidence intervals (CIs) for obesity, hypertension, high cholesterol, and high-risk lipid profiles. Missing WAHS data for individual items ranged from none to 11%, and 28% of participants had missing data on at least 1 item used in the analyses. To minimize bias, because missingness may not have been random and may have been associated with exposure or outcome or both, we used multiple imputation to impute values for missing WAHS data (17). This process used sequential regression multivariate imputation (18), as implemented in IVEware (University of Michigan, Ann Arbor, Michigan). In WA-BRFSS, missing data on items used in the analyses ranged from none to 11%, and 17% of participants had missing data on at least 1 item. For WA-BRFSS, participants with missing values on a variable were omitted from that analysis, because there were fewer missing data and WA-BRFSS does not use imputation for routine surveillance. Major analyses used Fisher’s *z* tests to compare WAHS and WA-BRFSS data and generalized estimating equations to conduct tests of paired comparisons between WAHS self-reported and examination-based findings. We used Wald χ^2^ tests to test demographic differences in sample characteristics between WAHS and WA-BRFSS. All analyses accounted for the sample designs and used design weights based on probability of selection and poststratification weights based on the age and sex distributions of the Washington State population. Statistical significance was set at *P* < .05.

WAHS did not measure self-reported obesity. This study conducted a Poisson regression to ensure that the difference between prevalence estimates of obesity based on physical measurements in WAHS and self-reports in WA-BRFSS was not due to demographic differences between the 2 samples. This analysis estimated a prevalence ratio for obesity in WAHS compared with WA-BRFSS, controlling for sex, age, race/ethnicity, education, annual household income, marital status, and household size. These analyses used the unimputed WAHS data because they required a combined WAHS and WA-BRFSS data set, and multiple imputation was not conducted for WA-BRFSS. Similar analyses compared self-reported hypertension and high cholesterol prevalence estimates from WAHS and WA-BRFSS.

We also developed density plots to depict the distributions of measured heights and weights in WAHS and self-reported values in WA-BRFSS.

## Results

### Sample characteristics

Distributions by sex, age, income, marital status, and household size were similar in the weighted WAHS and WA-BRFSS samples. The samples differed in distributions by race/ethnicity and education (Table 1).

**Table 1:** Sample Characteristics for 2006–2007 Washington Adult Health Survey (WAHS) and 2007 Washington Behavioral Risk Factor Surveillance System (WA-BRFSS)

Characteristic	WAHS		WA-BRFSS^a^	
	n	Weighted % (95% CI)	n	Weighted % (95% CI)
**Sex **				
Female	393	50.9 (45.5–56.3)	15,325	50.4 (49.6–51.3)
Male	279	49.1 (43.7–54.5)	9,321	49.6 (48.7–50.4)
**Age, y **				
25–39	195	29.3 (24.4–34.7)	4,357	30.6 (29.7–31.4)
40–59	305	45.3 (40.0–51.0)	10,300	43.9 (43.1–44.7)
≥60	172	25.5 (21.0–30.5)	9,989	25.5 (24.9–26.2)
**Race/ethnicity^b,c^**				
American Indian or Alaska Native, non-Hispanic	11	0.9 (0.4–2.2)	380	1.6 (1.4–1.9)
Asian, non-Hispanic	35	7.4 (3.9–13.9)	447	2.9 (2.6–3.3)
Black, non-Hispanic	29	3.8 (2.3–6.5)	318	1.9 (1.7–2.2)
Hispanic	91	9.5 (6.4–13.7)	1,036	6.1 (5.7–6.7)
Native Hawaiian or other Pacific Islander, non-Hispanic	3	0.3 (0.1–0.9)	85	0.6 (0.4–0.8)
White, non-Hispanic	497	78.1(71.5–83.6)	22,011	86.8 (86.1–87.4)
**Education^d^**				
≤High school graduate	247	30.5 (25.4–36.1)	7,462	28.0 (27.3–28.8)
Some college or technical school	242	37.2 (31.7–43.2)	7,826	30.6 (29.8–31.4)
≥ College graduate	183	32.3 (25.7–39.6)	9,283	41.4 (40.6–42.2)
**Annual household income, $ **				
<35,000	298	28.0 (22.8–33.9)	7,716	27.3 (26.6–28.1)
≥35,000	374	77.0 (70.5–82.5)	14,351	72.7 (71.9–73.4)
**Marital status **				
Married	339	68.0 (61.2–74.0)	14,585	69.0 (68.3–69.8)
Divorced	120	11.6 (8.8–15.2)	3,775	10.3 (9.8–10.8)
Widowed	56	4.8 (3.0–7.6)	3,009	5.8 (5.5–6.1)
Separated	28	2.0 (1.0–3.9)	435	1.5 (1.3–1.7)
Never married	85	7.4 (5.3–10.3)	2,048	9.4 (8.9–10.0)
Member of unmarried couple	46	6.2 (4.0–9.3)	705	4.0 (3.7–4.4)
**Household size, n **				
1	186	13.8 (11.1–16.9)	7,034	14.3 (13.9–14.8)
2	216	37.3 (31.5–43.5)	9,784	36.6 (35.8–37.4)
3	95	15.5 (12.7–18.9)	3,029	17.7 (17.0–18.4)
4	84	15.5 (11.9–20.0)	2,878	18.5 (17.8–19.2)
5	58	10.4 (7.5–14.1)	1,217	8.0 (7.4–8.5)
≥6	33	7.5 (4.8–11.6)	650	4.9 (4.5–5.4)
**Obesity/overweight^e^**				
Obese (BMI ≥30)	277	39.2 (33.6–45.1)	6,501	27.1 (26.3–27.8)
Overweight (25 ≤ BMI < 30)	230	37.5 (32.1–43.2)	8,694	37.7 (36.9–38.6)
Not overweight or obese (BMI <25)	165	23.4 (18.5-29.0)	8,360	35.2 (34.4–36.1)

Abbreviations: BMI, body mass index (kg/m^2^).

^a^ Includes nonpregnant participants aged 25 years or older to allow comparison with WAHS data.

^b^ Racial groups include participants who reported a single race only and participants who reported more than 1 racial group but provided a single race when asked which one of the groups best represented their race.

^c^ For race/ethnicity, Wald χ^2^ = 14.2 (*P* = .007) for overall comparison between WAHS and WA-BRFSS. Pacific Islanders were combined with Asians for this comparison because of their small numbers in WAHS.

^d^ For education, Wald χ^2^ = 7.2 (*P* = .03) for overall comparison between WAHS and WA-BRFSS.

^e^ WAHS estimates derived from physical measurements of height and weight; WA-BRFSS from self-report.

### Obesity

Approximately 39.2% of WAHS participants were classified as obese based on measured heights and weights, compared with 27.1% of WA-BRFSS participants based on self-reported heights and weights (*z* = 4.17, *P* < .001) (Table 1). Controlling for demographic differences did not change this finding. The prevalence ratio for obesity adjusted for demographic factors in WAHS compared with WA-BRFSS was 1.4 (95% CI, 1.2–1.6).

WAHS examination-based measurement of height and weight resulted in shorter heights and heavier weights (Figure), and higher BMIs, compared with WA-BRFSS. Adjusting for demographic factors, the ratios of mean heights and mean weights in WAHS compared with WA-BRFSS were 0.989 (95% CI, 0.986–0.993) and 1.043 (95% CI, 1.021–1.065), respectively.

**Figure:**
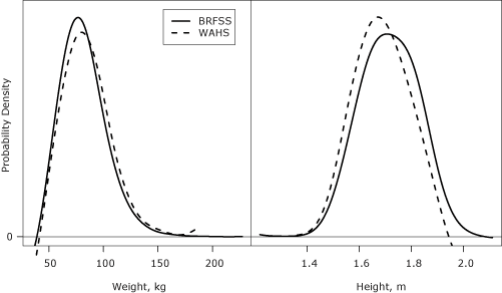
Comparison of the distributions of height and weight measurements from WAHS with self-reported heights and weights from WA-BRFSS. The vertical axis displays the probability density of the distributions. Abbreviations: WAHS, Washington Adult Health Survey; WA-BRFSS, Washington State Behavioral Risk Factor Surveillance System.

### Hypertension

Although prevalence estimates of hypertension derived from self-reports in WAHS (33.4%) and WA-BRFSS (28.1%) (Table 2) were significantly different (*z* = 2.52, *P* = .01), neither estimate differed significantly from the WAHS primary (29.4%) or secondary (31.1%) examination-based estimate. The higher prevalence estimate for self-reported hypertension in WAHS compared with WA-BRFSS persisted after controlling for demographic factors: the prevalence ratio for self-reported hypertension adjusted for demographic factors in WAHS compared with WA-BRFSS was 1.2 (95% CI, 1.1–1.4).

**Table 2:** Prevalence Estimates of Hypertension in the 2006–2007 Washington Adult Health Survey (WAHS) and the 2007 Washington Behavioral Risk Factor Surveillance System (WA-BRFSS)

Characteristic	n	Weighted % (95% CI)
**Self-reported hypertension^a^**		
WAHS	221	33.4 (29.4–37.7)
WA-BRFSS^b^	8,619	28.1 (27.4–28.8)
**WAHS only**		
**Examination-based hypertension**		
Primary measure (includes participants classified as definite or probable)	200	29.4 (25.8–33.4)
Secondary measure^c^	207	31.1 (27.3–35.1)
**Hypertension classification^d^**		
Definite	101	14.6 (11.7–18.2)
Probable	99	14.8 (12.2–17.7)
Possible	26	4.3 (2.6–7.1)
Probable no	37	6.1 (4.1–9.0)
Definite no	409	60.1 (55.9–64.3)

Abbreviation: CI, confidence interval.

^a^ Includes participants who reported that a health care provider had ever told them that they had high blood pressure.

^b^ Includes nonpregnant participants aged 25 years or older to allow comparison with WAHS data.

^c^ Similar to the measure used in the National Health and Nutrition Examination Survey (16): measured systolic blood pressure of 140 mm Hg or more, measured diastolic blood pressure of 90 mm Hg or more, or self-reported use of medication to control blood pressure based on answering yes to questions asking whether participants had been told to take prescribed medicine and whether they were following this advice.

^d^ Categories are mutually exclusive; participants classified at higher levels of certainty were not considered for lower levels. See Methods section for definitions of each category.

Approximately 1 in 20 (4.6%; 95% CI, 2.9%–7.1%) WAHS participants had possible undiagnosed hypertension. The sensitivity and specificity of self-reports compared with examination-based hypertension were 78% (95% CI, 70%–85%) and 85% (95% CI, 81%–89%), respectively.

### High cholesterol and other abnormal lipid values

Self-reported prevalence estimates of high cholesterol in WAHS (41.8%) and WA-BRFSS (38.3%) were similar (*z* = 1.13, *P* = .26) (Table 3). Both estimates were lower than the WAHS examination-based prevalence estimate of high-risk lipid profiles (WAHS, Wald *F*_1,50_ = 30.4, *P* < .001; WA-BRFSS, *z* = 8.25, *P* < .001). Controlling for demographic differences did not change the similarity of the WAHS and WA-BRFSS estimates based on self-reports: the prevalence ratio for self-reported high cholesterol adjusted for demographic factors in WAHS compared with WA-BRFSS was 1.1 (95% CI, 0.9–1.3). Approximately half (57.2%; 95% CI, 52.4%–61.8%) of participants had either borderline or high total cholesterol or were on cholesterol-lowering medication; approximately two-fifths self-reported high cholesterol; and about one-third had measured high cholesterol or were on cholesterol-lowering medication (29.2%; 95% CI, 25.2%–33.5%), so that the estimate of participants who self-reported high cholesterol was between the estimate for high cholesterol and the estimate for borderline or high cholesterol combined. 

**Table 3:** Prevalence Estimates of High Cholesterol and Other Abnormal Lipid Values, 2006–2007 Washington Adult Health Survey (WAHS) and the 2007 Washington Behavioral Risk Factor Surveillance System (WA-BRFSS)

Characteristic	n	Weighted % (95% CI)
**Self-reported high cholesterol^a^**		
WAHS	219	41.8 (35.8–48.1)
WA-BRFSS^b^	9,102	38.3 (37.5–39.2)
**WAHS only**		
**Examination-based lipids, mg/dL **		
Total cholesterol		
<200	388	57.7 (52.4–62.8)
200–239	196	29.4 (25.3–33.9)
≥240	89	12.9 (9.4–17.3)
Low-density lipoprotein cholesterol ≥160	70	10.9 (7.6–15.5)
Very low-density lipoprotein cholesterol ≥40	109	16.4 (13.2–20.2)
High-density lipoprotein cholesterol		
<40 for men and <50 for women	269	37.8 (33.2–42.5)
<40 for men and <40 for women	167	24.8 (20.8–29.3)
Triglycerides ≥200	123	18.3 (15.1–22.1)
**Examination-based high-risk lipid profile** (includes participants classified as definite or probable)	390	59.2 (54.2–64.2)
**High-risk lipid profile classification^c^**		
Definite	97	17.1 (13.6–21.3)
Probable	293	42.1 (37.3–47.2)
Possible	169	25.4 (21.3–29.9)
Probable no	5	0.3 (0–1.6)
Definite no	108	15.1 (11.9–19.0)

Abbreviation: CI, confidence interval.

^a^ Includes participants who reported that a health care provider had ever told them that they had high cholesterol. Excludes participants who said they had not had their cholesterol checked.

^b^ Includes nonpregnant participants aged 25 years or older to allow comparison with WAHS data.

^c^ Categories are mutually exclusive; participants classified at higher levels of certainty were not considered for lower levels. See Methods section for definitions of each category.

This study identified 28.1% (95% CI, 23.4%–33.4%) of WAHS participants who had possible undiagnosed high-risk lipid profiles, including 18.1% (95% CI, 14.4%–22.3%) who reported having their cholesterol tested and 10.1% (95% CI, 7.2%–14.0%) who reported never having been tested. Hispanic participants (43.0%; 95% CI, 31.2%–55.7%) were more likely than non-Hispanic participants (26.4%; 95% CI, 21.4%–32.1%) (*z* = 2.45, *P* = .01) and participants who did not graduate from college (31.8%; 95% CI, 26.4%–37.8%) were more likely than college graduates (20.4%; 95% CI, 12.4%–31.6%) (*z* = 2.05, *P* = .04) to have possible undiagnosed high-risk lipid profiles.

## Discussion

The prevalence of obesity estimated from WA-BRFSS self-reported heights and weights was significantly lower than that estimated from WAHS measured heights and weights. This finding did not appear to be due to demographic differences between the samples; the finding remained significant and of a similar magnitude after controlling for demographic factors. Although this finding is consistent with those of other studies suggesting that estimates of obesity based on self-reported heights and weights are underestimates (4-7), the magnitude of underestimation varies. Age, sex, BMI, race/ethnicity, and whether the person expects to be measured after self-report can affect the accuracy of self-reported heights and weights (7). 

The prevalence estimates of hypertension based on self-reports were not significantly different from the primary or secondary examination-based measure used in this study for WAHS data. Thus, routinely collected self-reported hypertension data in WA-BRFSS seem to provide a reasonable estimate of the overall prevalence of hypertension in Washington State. This finding contrasts with findings from studies in New York City and Australia. Compared with examination-based measures, the New York City study found that self-reported hypertension overestimated prevalence (6); the Australian study found that self-reports underestimated prevalence (9). The cause of these discrepant findings is unclear. Washington State’s small proportion of people who have possible undiagnosed hypertension may contribute to the stability of the Washington State estimate. Differences in findings between WAHS and other studies suggest using caution in generalizing from studies conducted in different geographic areas or among populations that are not demographically similar.

Prevalence estimates of high cholesterol based on self-reports were significantly lower than those for high-risk lipid profiles based on WAHS examinations. Hispanics (compared with non-Hispanics) and participants who did not graduate from college (compared with college graduates) were significantly more likely to have possible undiagnosed high-risk lipid profiles. These groups were also less likely to report having had their cholesterol checked in the 2007 WA-BRFSS. Thus, the WA-BRFSS may have limited value in identifying both the proportion of the population with high-risk lipid profiles and subgroups at higher risk than the general population.

Of WAHS participants who had possible undiagnosed high-risk lipid profiles and who said they had been tested, approximately half had low HDL cholesterol as the only abnormal lipid. They could have accurately answered no to the question on high cholesterol even if they were aware of their HDL status. Other reasons for discrepancies among self-reported and examination-based hypertension, high cholesterol, or high-risk lipid profiles include anomalous readings in WAHS, recently developed conditions, incorrect recall, and control of the condition through behavioral approaches, such as physical activity or diet. One study of cholesterol recall reported that 89% of participants accurately remembered their risk category (normal, borderline, or high) for periods of 1 to 6 months when they were consistently counseled by providers who used these categories and the same categories were reflected in the recall measure (19). Information about how health care providers in Washington counsel patients about their blood pressure or cholesterol levels is not available.

Although differences in education and race/ethnicity between the WAHS and WA-BRFSS samples potentially limit the validity of our comparisons, findings persisted when we controlled for demographic factors. The low response rate also potentially limits the validity of this study. However, several recent reviews found little relationship between response rates and the amount of nonresponse bias. The range of response rates in the studies in those reviews was approximately 25% to 85% (20–22). The different methods used by WAHS and WA-BRFSS may attract study participants differing on unmeasured characteristics. One strength of this study is its novel approach of differentiating levels of diagnostic certainty for examination-based hypertension and high-risk lipid profiles.

As the role of individual components of cholesterol and triglycerides in identifying people at high risk for cardiovascular disease becomes more established, the BRFSS question measuring high cholesterol may be less helpful in identifying people at high risk for cardiovascular disease. Research into how physicians explain lipid values to patients; how patients hear, interpret, and recall these messages; and how study participants interpret survey questions on blood lipids may aid in developing questions that facilitate accurate self-reporting of high-risk lipid profiles. Periodic examination-based measurement provides perspective on routinely collected self-reports.

## Figures and Tables

**Table:** 

**1. The activity supported the learning objectives.**				
**Strongly Disagree**				**Strongly Agree**
1	2	3	4	5
**2. The material was organized clearly for learning to occur.**				
**Strongly Disagree**				**Strongly Agree**
1	2	3	4	5
**3. The content learned from this activity will impact my practice.**				
**Strongly Disagree**				**Strongly Agree**
1	2	3	4	5
**4. The activity was presented objectively and free of commercial bias.**				
**Strongly Disagree**				**Strongly Agree**
1	2	3	4	5

## Post-Test Information

To obtain credit, you should first read the journal article. After reading the article, you should be able to answer the following, related, multiple-choice questions. To complete the questions (with a minimum 70% passing score) and earn continuing medical education (CME) credit, please go to http://www.medscape.org/journal/pcd. Credit cannot be obtained for tests completed on paper, although you may use the worksheet below to keep a record of your answers. You must be a registered user on Medscape.org. If you are not registered on Medscape.org, please click on the "Register" link on the right hand side of the website to register. Only one answer is correct for each question. Once you successfully answer all post-test questions you will be able to view and/or print your certificate. For questions regarding the content of this activity, contact the accredited provider, CME@medscape.net. For technical assistance, contact CME@webmd.net. American Medical Association's Physician's Recognition Award (AMA PRA) credits are accepted in the US as evidence of participation in CME activities. For further information on this award, please refer to http://www.ama-assn.org/ama/pub/category/2922.html. The AMA has determined that physicians not licensed in the US who participate in this CME activity are eligible for **AMA PRA Category 1 Credits™**. Through agreements that the AMA has made with agencies in some countries, AMA PRA credit may be acceptable as evidence of participation in CME activities. If you are not licensed in the US, please complete the questions online, print the AMA PRA CME credit certificate and present it to your national medical association for review.

## Post-Test Questions

### Article Title: Examination-Based vs Self-Reported Risk Factors for CVD

**CME Questions**

1. You are interpreting results of community questionnaires regarding cardiovascular risk factors. Based on the results of the current study, what should you consider regarding the results of self-reported vs examination-based data regarding obesity?
  - There is no significant difference between self-reported and examination-based data
  - The rate of obesity is higher in self-reported vs examination-based data
  - The rate of obesity is higher in examination-based vs self-reported data
  - Self-reported data on body weight was lower than examination-based weight data, but data on height was the same
2. What was the relationship between self-reported and examination-based data regarding hypertension in the current study?
  - There was no significant difference between self-reported and examination-based data
  - The rate of hypertension was higher in self-reported vs examination-based data
  - The rate of hypertension was higher in examination-based vs self-reported data
  - The rate of hypertension was higher in examination-based vs self-reported data only among men
3. What should you consider regarding the relationship between rates of self-reported and examination-based hyperlipidemia in the current study?
  - There was no significant difference between self-reported and examination-based data
  - Rates of self-reported hyperlipidemia were higher vs examination-based hyperlipidemia
  - Rates of examination-based hyperlipidemia were higher vs self-reported hyperlipidemia
  - Only low-density lipoprotein cholesterol values were different in comparing self-reported and examination-based data
4. Which of the following variables was most associated with undiagnosed hyperlipidemia in the current study?
  - Obesity
  - Hispanic ethnicity
  - Male sex
  - Living in a rural area

**Evaluation**
